# Therapeutic Targeting of CDK7 Suppresses Tumor Progression in Intrahepatic Cholangiocarcinoma

**DOI:** 10.7150/ijbs.39779

**Published:** 2020-02-10

**Authors:** Hua-Dong Chen, Chen-Song Huang, Qiong-Cong Xu, Fuxi Li, Xi-Tai Huang, Jie-Qin Wang, Shi-Jin Li, Wei Zhao, Xiao-Yu Yin

**Affiliations:** 1 Department of Pancreato-Biliary Surgery, The First Affiliated Hospital of Sun Yat-sen University, Guangzhou 510080, China; 2 Department of Pediatric Surgery, The First Affiliated Hospital of Sun Yat-sen University, Guangzhou 510080, China; 3 RNA Biomedical Institute, Sun Yat-Sen Memorial Hospital, Sun Yat-Sen University, Guangzhou 510120, China; 4 Key Laboratory of Stem Cells and Tissue Engineering (Sun Yat-Sen University), Ministry of Education, Guangzhou 510080, China

**Keywords:** Intrahepatic cholangiocarcinoma, Cyclin-dependent Kinase 7, THZ1, Cell cycle, c-Met

## Abstract

Intrahepatic cholangiocarcinoma (ICC) is a lethal malignancy with high mortality and lack of effective therapeutic targets. Here, we found that expression of cyclin-dependent kinase 7 (CDK7) was significantly associated with higher tumor grade and worse prognosis in 96 ICC specimens. Depletion of CDK7 significantly inhibited cell growth, induced a G2/M cell cycle arrest, and reduced the migratory and invasive potential in ICC cells. Subsequent experiments demonstrated that ICC cells were highly sensitive to the CDK7 inhibitor THZ1. A low concentration of THZ1 markedly inhibited cell growth, cell cycle, migration, and invasion in ICC cell lines. RNA-sequencing (RNA-seq) analysis revealed that THZ1 treatment decreased the levels of massive oncogene transcripts, particularly those associated with cell cycle and cell migration. Quantitative reverse transcriptase PCR (qRT-PCR) analysis confirmed that transcription of oncogenes involved in cell cycle regulation (*AURKA, AURKB, CDC25B, CDK1*, *CCNA2*, and *MKI67*) and the c-Met pathway (*c-Met, AKT1, PTK2, CRK, PDPK1*, and *ARF6*) was selectively repressed by THZ1. In addition, THZ1 exhibited significant anti-tumor activity in a patient-derived xenograft (PDX) model of ICC, without causing detectable side effects.

## Introduction

Intrahepatic cholangiocarcinoma (ICC) is the second most common primary malignant liver tumor after hepatocellular carcinoma (HCC)^1^. While curative resection is the most effective treatment for ICC patients, two-thirds of the tumors are already locally advanced or metastatic at the time of diagnosis, and thus not amenable to resection 2. Patients with unresectable ICC receiving standard palliative chemotherapy (gemcitabine and cisplatin) have a median survival of less than one year 3. The scarcity of treatment options and poor prognosis necessitate the discovery of novel therapeutic targets for ICC.

Members of the cyclin-dependent kinase (CDK) family play central regulatory roles in cell cycle progression and gene transcription, and are potential therapeutic targets in various types of cancer 4. CDK7 is a major member of the CDK family. Together with cyclin H and MAT1, CDK7 forms the trimeric complex CDK-activating kinase (CAK), whose role is to phosphorylate other CDKs 5. CDK7 controls both the G2/M and G1/S cell cycle transitions. Moreover, as an essential component of the transcription factor TFIIH, CDK7 is involved in transcription initiation by phosphorylating RNA polymerase II (RNAPII) 6. Inhibition of CDK7 activity was shown to impair transcription and cell cycle progression, and suppress tumor growth 7, suggesting that CDK7 could serve as a therapeutic target in ICC.

The early-generation CDK inhibitors, which were non-selective and targeted multiple CDKs, exhibited modest anti-tumor activity and unfavorable toxicity profiles in late-stage clinical trials 8. THZ1 is a highly specific CDK7 inhibitor with an acrylamide moiety that selectively binds to the cysteine 312 residue on CDK7. Recent studies have shown that THZ1 regulates RNAPII phosphorylation and controls transcriptional initiation, pausing, and elongation 6, 9, 10. THZ1 exhibits potent anti-tumor activity in several cancer types, including small cell lung cancer 11, ovarian cancer 12, and triple-negative breast cancer 13, but its effects on ICC have not yet been elucidated.

In this study, we used immunohistochemical (IHC) staining to show that elevated CDK7 expression correlated with worse clinical outcomes and poor prognosis in ICC patients. Moreover, we used small interfering RNAs (siRNAs) and THZ1 to impair CDK7 activity *in vitro*. CDK7 inhibition induced a G2/M cell cycle arrest, reduced cell growth, and decreased the migratory and invasive ability in ICC cells. RNA-seq analysis revealed that the therapeutic effect of THZ1 in ICC was mediated via transcriptional repression of genes associated with cell cycle progression and the c-Met signaling pathway. We also investigated whether THZ1 could inhibit ICC tumor growth *in vivo*.

## Materials and Methods

### ICC specimens and IHC staining

A total of 96 ICC specimens were obtained from patients who underwent surgical resection of ICC at the First Affiliated Hospital of Sun Yat-sen University (Guangzhou, China) between January 2003 and December 2006. The patient inclusion criteria were as follows: (1) underwent R0 resection; (2) received no preoperative chemotherapy; (3) had no distant metastases; (4) survived for over 30 days after the operation; (5) integrated clinical-pathological and follow-up data available. The tissue samples were embedded in paraffin and used for clinicopathological and prognostic analysis. The clinicopathological characteristics of the samples are summarized in **Table S1**. This study was approved by the Ethics Committee of the First Affiliated Hospital of Sun Yat-sen University.

Immunohistochemical (IHC) staining was performed as previously described 14. Two experienced pathologists independently scored the samples for the percentage of CDK-7-positive cells (IHC score) and staining intensity. The cut-off values for high and low protein expression were selected based on a measurement of heterogeneity using the log-rank test with respect to Overall Survival (OS) and Disease-Free Survival (DFS).

### Reagents and antibodies

THZ1 (S7549) was purchased from Selleck Chemicals. Small interfering RNA (siRNA) targeting human *CDK7* and a non-targeting control siRNA were purchased from Genepharma (Suzhou, China). The pReceiver-M98-CDK7 overexpression plasmid and pReceiver-M98 empty vector were purchased from Genecopoeia (Rockville, MD). The CDK7 antibody was purchased from ProteinTech Group (USA).

### Cell culture and transfection

Human ICC cell lines (RBE and SSP-25) were obtained from the General Surgery Laboratory of the First Affiliated Hospital of Sun Yat-sen University. The cells were cultured in RPMI 1640 medium supplemented with 10% fetal bovine serum (FBS), and maintained at 37 °C in a humidified incubator with 5% CO_2_. Transient transfection of siRNA or plasmids was performed according to the manufacturers' protocols, as described previously 14. The sequences of primers and siRNAs used in this study are listed in **Table S2**.

### Quantitative real-time PCR (qRT-PCR)

The procedure for qRT-PCR has been described previously 14. Briefly, the total RNA was extracted using TRIzol reagent (Life Technologies, USA) according to the manufacturer's instructions. The RNA was reverse-transcribed to cDNA using the Maxima First Strand cDNA Synthesis Kit for RT-PCR (Thermo Scientific^TM^, USA). The qRT-PCR assay was performed on a QuantStudio 6 Flex Real-time PCR system using the Takana SYBR^®^ Primix Ex TaqTM Kit (Takana, Dalian, China).

### Cell viability and calculation of half-maximal inhibitory concentration (IC50)

The cells were seeded in 96-well plates in 100 μL RPMI 1640 medium containing 10% FBS, at a density of 4×10^3^ cells per well. The cells were exposed to different concentrations of THZ1 and assayed for viability at 24, 48, and 72 h post-treatment, using the CellTiter-Glo Luminescent Cell Viability Assay (Promega) according to the manufacturer's instructions. The absorbance values were normalized with respect to those of untreated control cells. The IC50 was calculated using non-linear regression analysis in GraphPad Prism 6.0.

### Cell cycle assay

The cells were treated with THZ1 or CDK7 siRNA for 48 h, then harvested, rinsed with phosphate-buffered saline (PBS) at 4 °C, and fixed with 70% ice-cold ethanol for 30 minutes on ice. The fixed cells were incubated with propidium iodide (PI) from the Cell Cycle Staining Kit (CCS012; MultiSciences Biotech. Co.) for 30 minutes before detection. Flow cytometry data was acquired on a CytoFLEX cytometer (Beckman Coulter) and analyzed using CytExpert software.

### Cell invasion and migration assays

To evaluate cell migration, approximately 4×10^4^ cells in 300 μL RPMI 1640 medium without FBS were seeded into upper Transwell chambers (8 μm pore size). The lower chambers were filled with 800 μL RPMI 1640 medium supplemented with 10% FBS. After 24 h, the cells attached to the lower surface of the membrane were fixed with 4% formaldehyde, stained with 0.5% crystal violet, and then counted under a microscope in five random fields. Each experiment was done in triplicate. Invasion assays were performed under the same conditions as the migration assays, but in Matrigel (Corning, NY, USA)-coated Transwell inserts.

### Formation of ICC tumor spheroids

To form three-dimensional tumor spheroids, RBE and SSP-25 cells were seeded at a density of 2×10^3^ cells per 100 μL RPMI 1640 complete medium per well in a Corning^®^ 96-Well Ultra Low Attachment Microplate. After five days of incubation, the cells were photographed and counted under an inverted microscope.

### Patient-derived xenograft (PDX) model and THZ1 treatment

ICC PDX (PDX0044), with three passages in B-NDG^®^ mice (Biocytogen, Beijing, China), were inoculated subcutaneously into the right flanks of 4-week-old female BALB/c (nu/nu) nude mice. Tumor volume was calculated as length × width^2^/2. Once the xenografts reached a volume of 50-100 mm^3^, the mice were randomly divided into two groups and treated intraperitoneally with either PBS or THZ1 (10 mg/kg body weight) twice daily. Tumor volume was measured at 4-day intervals. After 17 days, the mice were euthanized under the guidance of Institutional Animal Care and Use Committee (IACUC) of Sun Yat-sen University. The concentration of serum alanine aminotransferase (ALT), aspartate aminotransferase (AST), and blood urea nitrogen (BUN) was measured. The tumor xenografts and organs were excised, fixed, weighed, photographed, and paraffin-embedded for hematoxylin and eosin (H&E) staining. All the animal experiments were carried out with the approval of the Institutional Review Board of the First Affiliated Hospital of Sun Yat-sen University ([2019] No. 124).

### RNA-seq preparation and analysis

The RNA sequencing (RNA-seq) was performed by Novogene (Beijing, China). An R package, DESeq, was used to quantify transcription levels and identify differentially expressed genes, using a cut-off of* P*<0.05. All heatmaps were built with R programming. The genes with |log2 (fold change)| > 1 and *P*<0.05 were used for gene ontology (GO) analysis via the clusterProfiler package in R software. Pathway enrichment analysis was conducted using Gene Set Enrichment Analysis (GSEA) software provided by the Broad Institute.

### Statistical analyses

All the experiments were repeated at least three times. The data was compared between the groups using unpaired Student's t-test or Chi square test. The continuous variables were expressed as mean ± SD. The DFS and OS were determined by the Kaplan-Meier method, and the differences between the groups were evaluated using the log-rank test. In all the statistical analyses, differences with *P*<0.05 were considered to be statistically significant.

## Results

### Elevated CDK7 expression was associated with poor clinical outcome in patients with ICC

**Figure 1:**
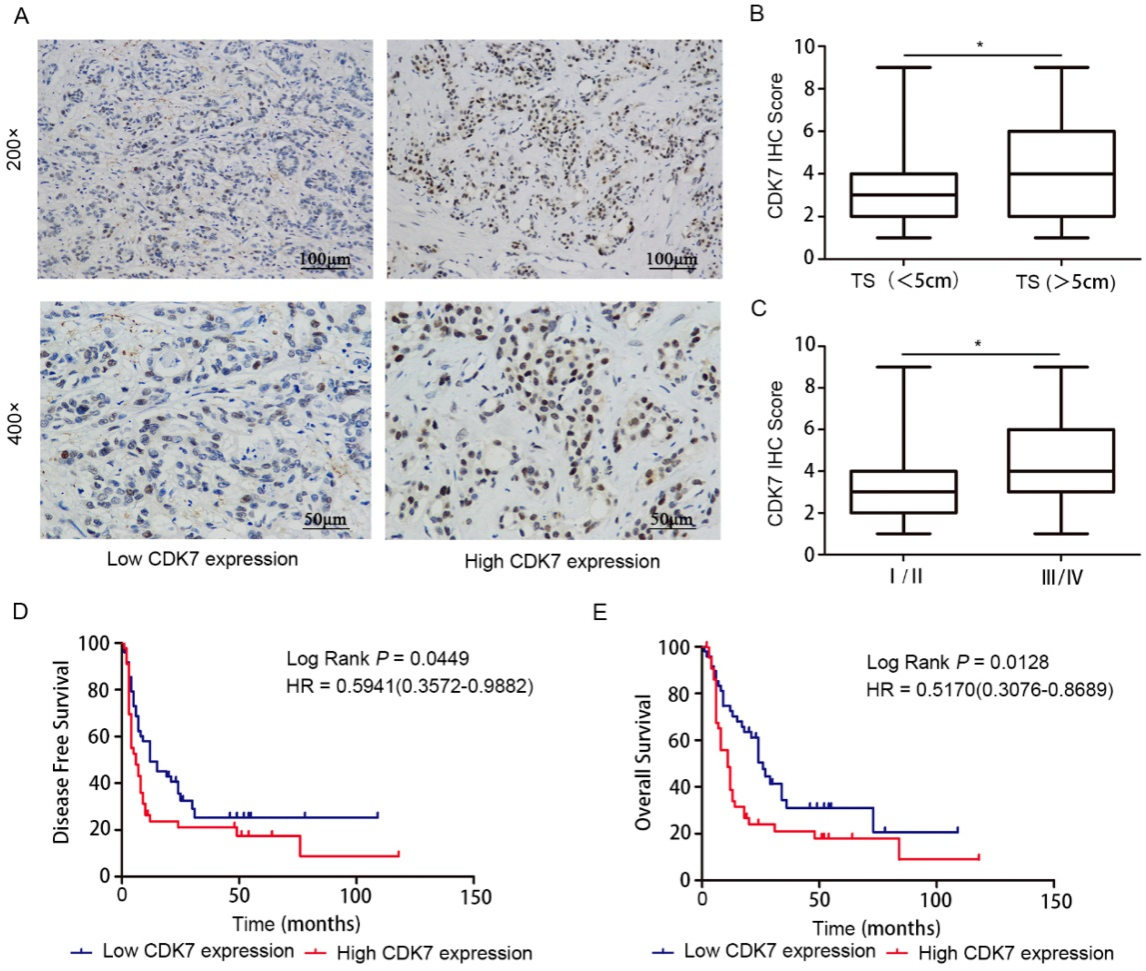
** Prognostic value of CDK7 expression in ICC.** A. Representative images of ICC tumors expressing low or high levels of CDK7, as determined by IHC. B. Correlation analysis of CDK7 expression with tumor size (TS; tumor diameter less than 5 cm vs. more than 5 cm). C. Correlation analysis of CDK7 expression with tumor TNM stage (stage I/II vs. III/IV). The boundaries of the box represent the 25th and 75th percentile of the data, while the horizontal line inside the box denotes the median value. D. Kaplan-Meier survival curves of DFS in 96 ICC patients, stratified by CDK7 IHC score (CDK7 low expression, n=51 vs. CDK7 high expression, n=45). The *P-*value was calculated using the log-rank test. HR, Hazard Ratio. E. Kaplan-Meier survival curves of OS in 96 ICC patients, stratified by CDK7 IHC score (CDK7 low expression, n=51 vs. CDK7 high expression, n=45). The *P-*value was calculated using the log-rank test. HR, Hazard Ratio.

To investigate whether CDK7 expression is associated with clinical outcome in ICC, we measured CDK7 protein level in ICC tissues (n=96) using IHC staining, and then correlated the findings with clinicopathologic variables. CDK7 was located in the nuclei of tumor cells (**Figure 1A**). High IHC score was significantly associated with increased tumor size (*P*<0.05)** (Figure 1B)** and tumor late-stage (*P*<0.05) **(Figure 1C)**. We observed no correlations between CDK7 expression and other parameters, such as patients' gender and age (**Table 1**).

Kaplan-Meier survival analysis demonstrated that, when using the median CDK7 IHC score as cut-off point for stratification, high CDK7 expression was strongly correlated with reduced DFS (*P*=0.0449) (**Figure 1D**) and OS (*P*=0.0128) (**Figure 1E**) in ICC. These findings indicate that CDK7 expression may predict survival outcome in ICC.

### Knockdown of CDK7 inhibited cell proliferation, cell growth, sphere formation efficiency, migration, and invasion of ICC cells *in vitro*

**Figure 2:**
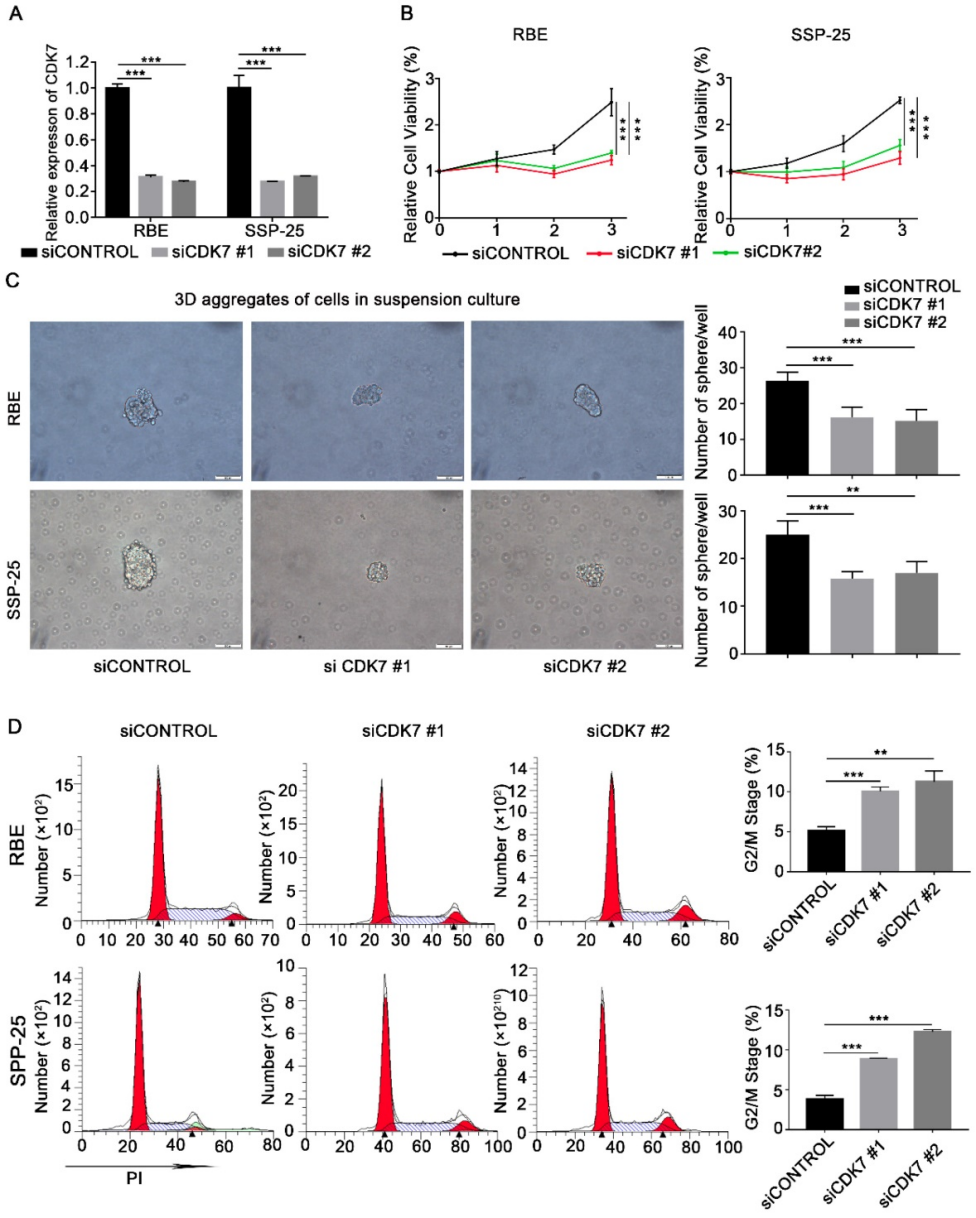
** CDK7 knockdown inhibited cell proliferation and blocked the cell cycle in ICC cells* in vitro*.** A. Relative expression of CDK7 mRNA in siControl and si*CDK7* cells (RBE and SSP-25 cells). B. Cell growth curve of RBE and SSP-25 cells upon *CDK7* siRNA transfection. C. Tumor sphere formation in RBE and SSP-25 cells following *CDK7* siRNA transfection for 48 h. The spheres were counted 5 days after siRNA transfection. Scale bar= 100 μm. D. Cell cycle analysis of RBE and SSP-25 cells transfected with *CDK7* siRNA or non-targeting control siRNA. The results are presented as mean ± SD of three independent experiments (*P<0.05, **P<0.01, ***P<0.001, according to a Student's t-test).

To investigate the functional effects of CDK7 on ICC cells, CDK7 expression in RBE and SSP-25 cells was suppressed by *CDK7* siRNA transfection. Efficient knockdown of *CDK7* was confirmed via qRT-PCR (**Figure 2A**). Cell growth was significantly reduced in both *CDK7* knockdown (si*CDK7*) cell lines compared with the control cells (siControl) (**Figure 2B**). Subsequently, we performed the sphere formation assay to investigate the effect of *CDK7* knockdown on the tumorigenicity of RBE and SSP-25 cells. The number of spheres was significantly lower in both si*CDK7* cell lines compared with their respective control cells (**Figure 2C**).

To further test the effect of *CDK7* knockdown on ICC cell growth, we used flow cytometry to analyze the cell cycle of si*CDK7* and siControl RBE and SSP-25 cells. Silencing of CDK7 expression caused a substantial accumulation of cells in the G2/M phase of the cell cycle (**Figure 2D**). These data suggest that *CDK7* knockdown suppresses ICC cell proliferation via induction of cell cycle arrest.

**Figure 3:**
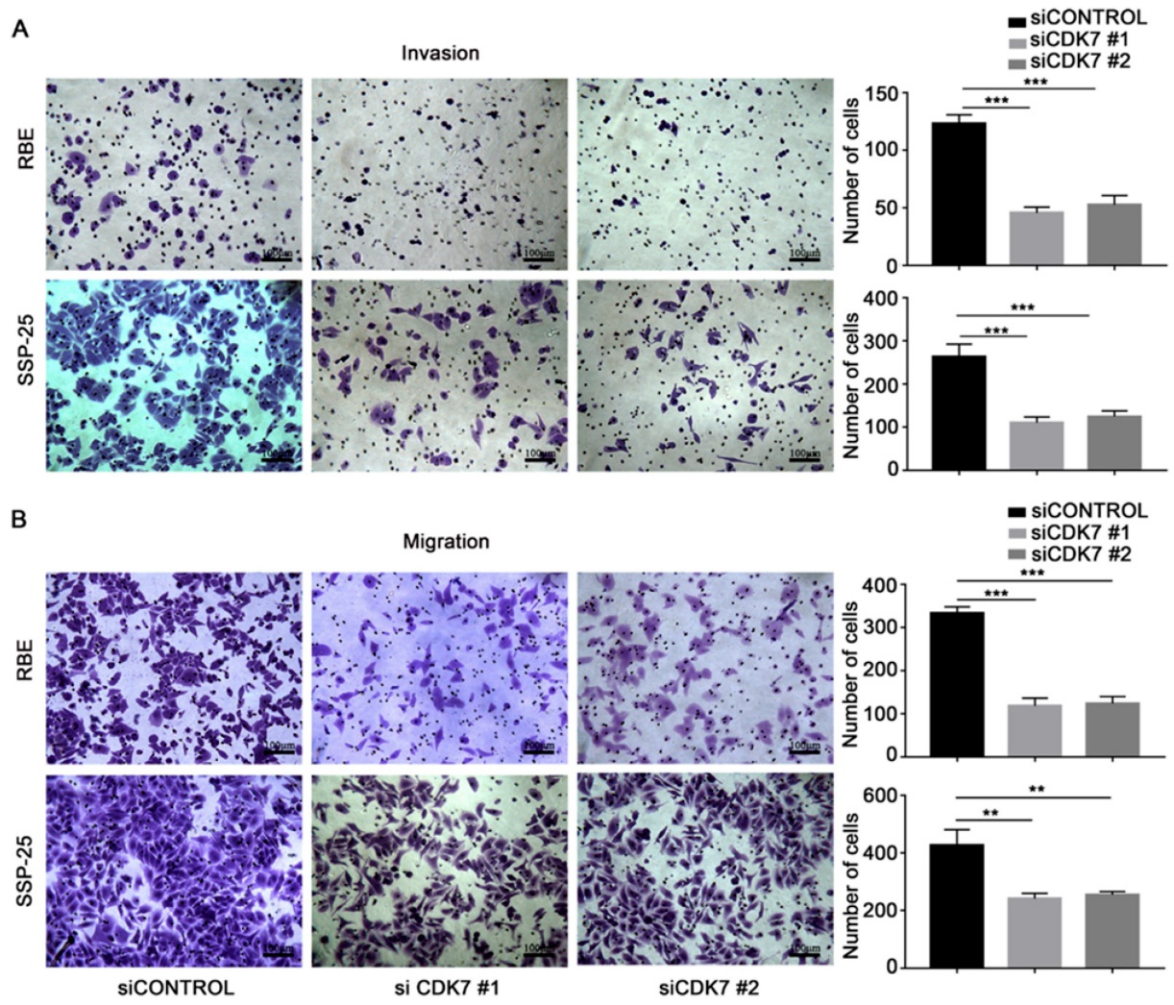
** CDK7 knockdown inhibited ICC cell invasion and migration *in vitro*.** A and B. The cells transfected with *CDK7* siRNA or non-targeting control siRNA for 48 h were used in the invasion (A) and migration (B) assay. The cells that reached the bottom of the membrane were photographed (200× magnification), extracted, and counted. The results are presented as mean ± SD of three independent experiments (*P<0.05, **P<0.01, ***P<0.001, according to a Student's t-test).

Next, we examined the effect of *CDK7* knockdown on the migration and invasion abilities of RBE and SSP-25 cells. Both si*CDK7* cell lines displayed decreased invasion (**Figure 3A**) and migration (**Figure 3B**) compared to their respective control cells.

### CDK7 inhibitor THZ1 exhibited a potent anti-tumor effect on ICC cells

**Figure 4:**
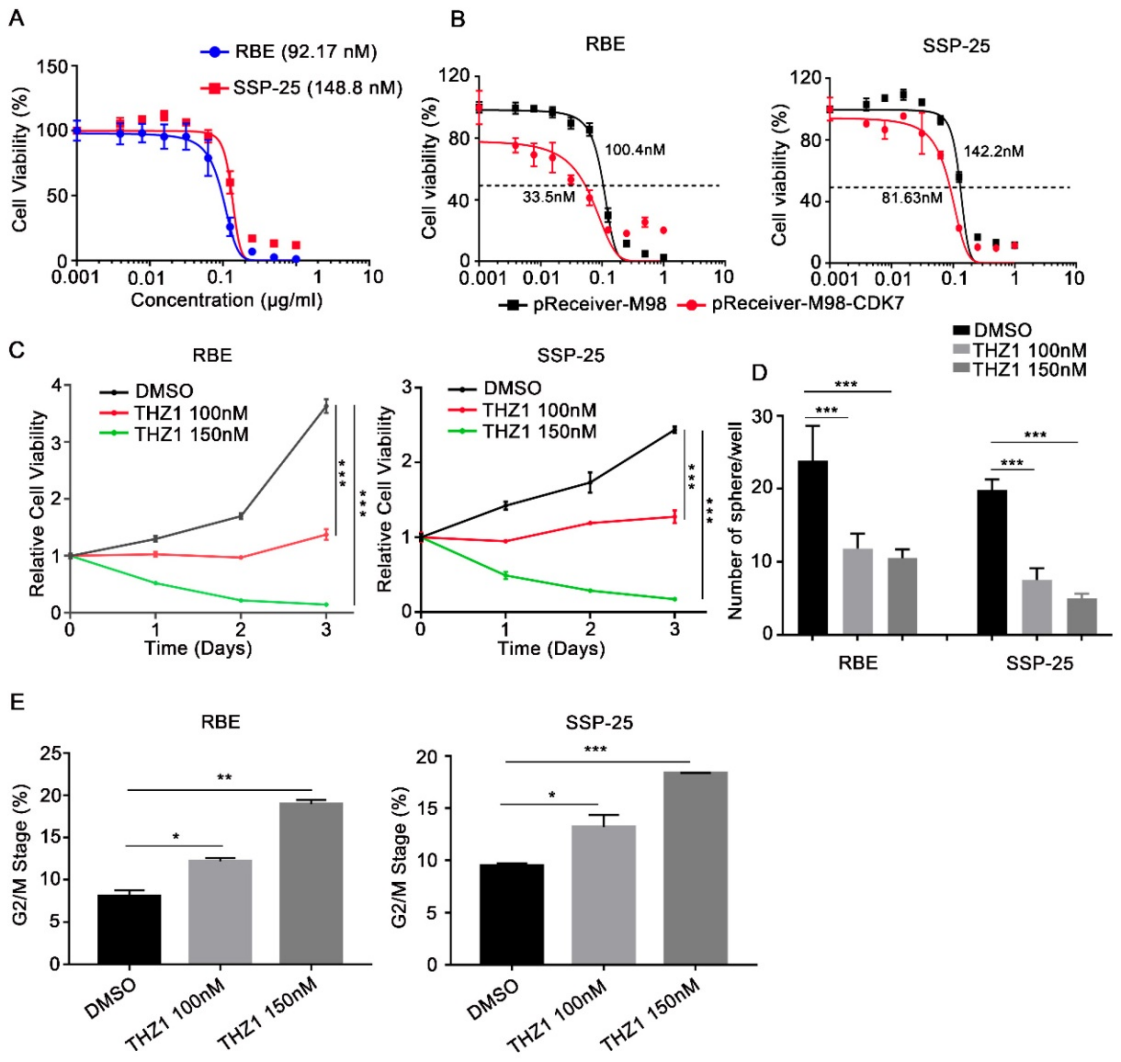
** CDK7 inhibitor THZ1 suppressed ICC cell proliferation and cell cycle progression *in vitro*.** A. RBE and SSP-25 cells were treated with the indicated concentrations of THZ1 for 48 h. Cell viability relative to that of DMSO-treated cells is shown. B. RBE and SSP-25 cells were transfected with *CDK7*-overexpressing vector (pReceiver-M98-CDK7) or control vector (pReceiver-M98) before they were treated with the indicated concentrations of THZ1 for 48 h. Cell viability relative to that of DMSO-treated cells is shown. C. Cell growth curve of RBE and SSP-25 cells upon treatment with different concentrations of THZ1 (100 nM or 150 nM) or 1‰ DMSO. D. Tumor sphere formation in RBE and SSP-25 cells upon treatment with different concentrations of THZ1 (100 nM or 150 nM) or 1‰ DMSO on day 5. The spheres were counted on day 7. E. Cell cycle analysis of RBE and SSP-25 cells upon treatment with different concentrations of THZ1 or 1‰ DMSO for 48 h. The results are presented as mean ± SD of three independent experiments (*P<0.05, **P<0.01, ***P<0.001, according to a Student's t-test).

THZ1, a recently identified covalent CDK7 inhibitor, displays high therapeutic potency in a variety of cancers. Here, we found that THZ1 also reduced cell viability in the two ICC cell lines (IC50=92.17 nM and 148.8 nM in RBE and SSP-25 cells, respectively) (**Figure 4A**). Moreover, ectopic expression of CDK7 increased the sensitivity of RBE and SSP-25 cells to THZ1 treatment (**Figure 4B**), indicating that THZ1 targets CDK7 in ICC cells.

**Figure 5:**
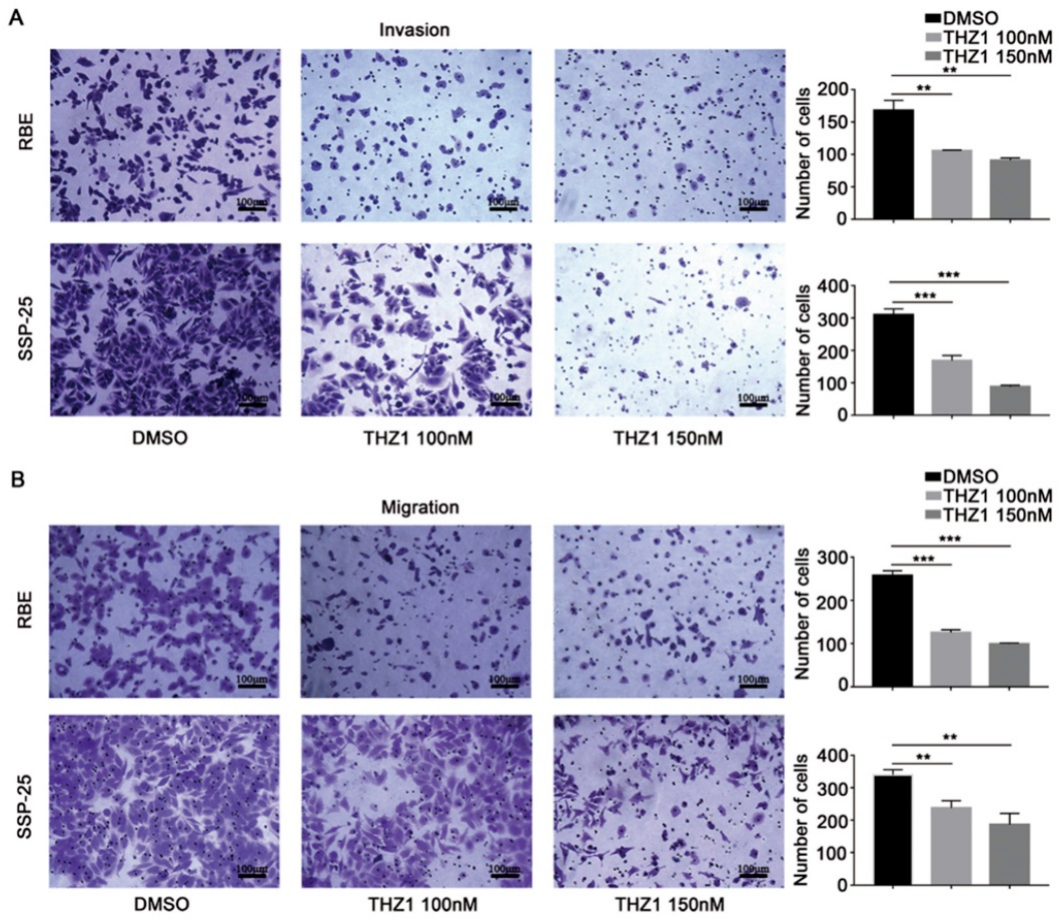
** CDK7 inhibitor THZ1 suppressed ICC cell migration, invasion, and tumor sphere formation *in vitro*.** A and B. Cells treated with THZ1 (100 nM or 150 nM) or 1‰ DMSO for 48 h were used in an invasion (A) and migration (B) assay. The cells that reached the bottom of the membrane were photographed (200× magnification), extracted and counted. The results are presented as mean ± SD of three independent experiments (*P<0.05, **P<0.01, ***P<0.001, according to a Student's t-test).

As expected, THZ1 reduced the proliferation of RBE and SSP-25 cells in a dose-dependent manner (**Figure 4C**). Under suspension cell culture conditions, the average number of colonies formed after exposure to THZ1 was significantly lower in ICC cells than in the control cells (**Figure 4D** and **Figure S1A**). Furthermore, THZ1 treatment induced a dose-dependent G2/M cell cycle arrest in both ICC cell lines (**Figure 4E** and **Figure S1B**), and inhibited cell invasion (**Figure 5A**) and migration (**Figure 5B**) in a dose-dependent manner (*P*<0.05, respectively).

### THZ1 inhibited ICC progression through blocking transcription of cell cycle and c-Met signaling pathway genes

**Figure 6:**
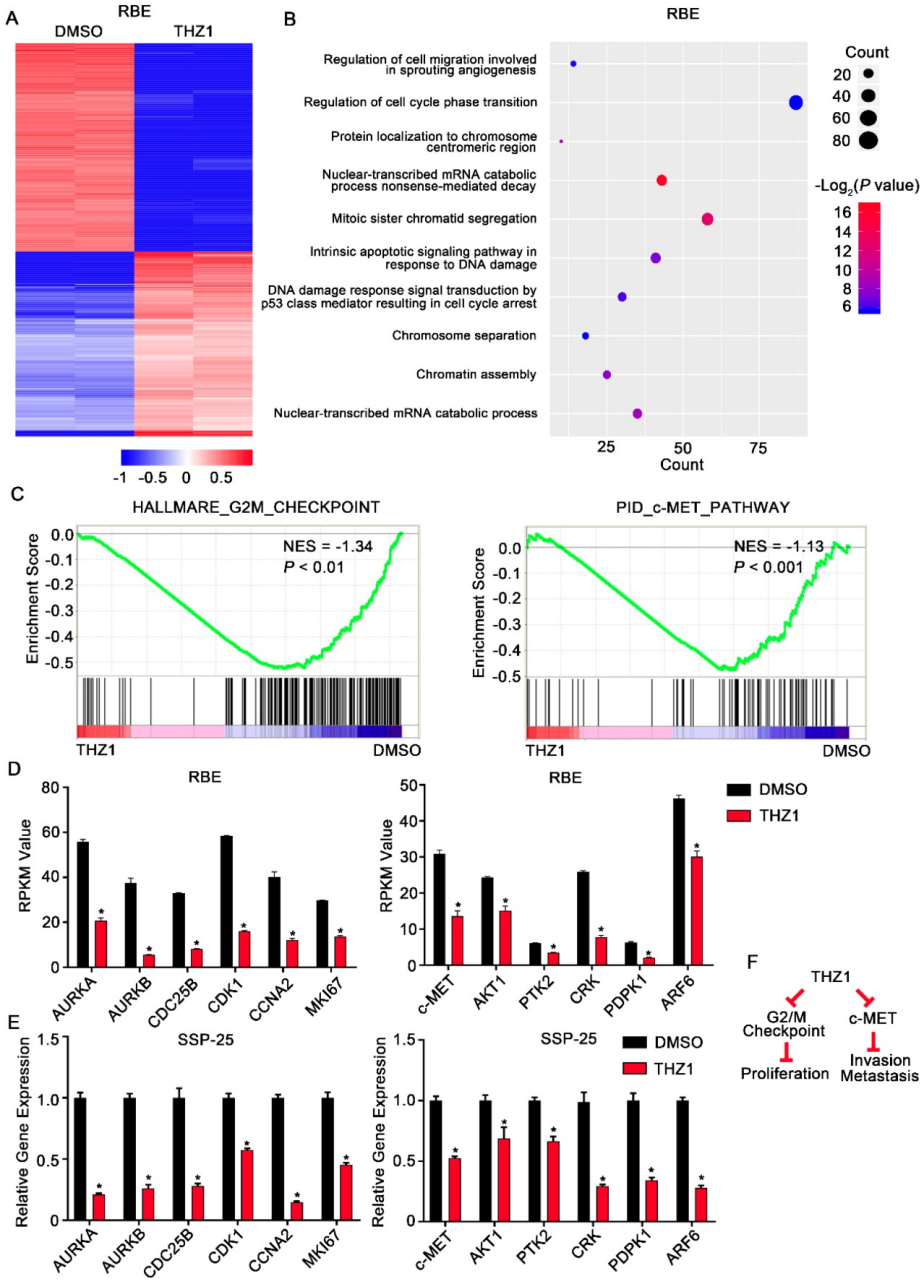
** THZ1 inhibited G2/M progression and c-Met signaling in ICC cells.** A. Heatmap of the differentially expressed genes (|log2 (fold change)| > 1 and *P*< 0.05) in RBE cells treated with 100 nM THZ1 or 1‰ DMSO for 24 h. The rows show Z-scores calculated for each gene. B. Gene ontology (GO) analysis of the differentially expressed genes (|log2 (fold change)| > 1 and *P*<0.05) in RBE cells. C. Gene set enrichment analysis (GSEA) revealed that G2/M checkpoint gene sets and c-Met pathway gene sets were enriched in RBE cells treated with 100 nM THZ1 for 24 h. D. Relative expression of *AURKA, AURKB, CDC25B, CDK1, CCNA2,* and* MKI67* in RBE cells treated with 100 nM THZ1 or 1‰ DMSO for 24 h. E. Relative expression of *c-Met, AKT1, PTK2, CRK, PDPK1,* and *ARF6* in RBE cells treated with 100 nM THZ1 or 1‰ DMSO for 24 h. F. Mechanistic scheme of the anti-ICC effects of THZ1. The results are presented as mean ± SD of three independent experiments (*P<0.05, **P<0.01, ***P<0.001, according to a Student's t-test).

To further delineate the molecular mechanisms underlying the effects of THZ1 on ICC, we conducted RNA-seq analysis to assess the genome-wide effect of THZ1 on gene expression in RBE cells (**Figure 6A**). Gene ontology (GO) analysis of the differentially expressed genes revealed a significant enrichment of genes involved in cell cycle regulation, cell invasion, and cell migration (**Figure 6B**). Moreover, GSEA showed that genes controlling the G2/M checkpoint, and genes associated with the c-Met pathway, were significantly enriched in the THZ1-downregulated transcripts (**Figure 6C and D**).

qRT-PCR confirmed that protein levels of the downregulated cell cycle genes, *AURKA, AURKB, CDC25B, CDK1, CCNA2,* and* MKI67*, were markedly reduced following THZ1 exposure (**Figure 6E**). Aurora kinases (Aurora A/B), CDC25B, and CDK1 control the G2/M checkpoint. Therefore, the increased G2/M arrest following THZ1 treatment was caused by decreased transcription of *AURKA, AURKB, CDC25B*, and* CDK1* genes (**Figure 6F, left**). Moreover, qRT-PCR confirmed that THZ1 impaired the transcription of c-Met signaling genes (*c-Met, AKT1, PTK2, CRK, PDPK1,* and *ARF6*) (**Figure 6E**). c-MET and its ligand, HGF, are frequently overexpressed in liver cancer and in associated metastases. Thus, the inhibitory effects of THZ1 on ICC cell migration and invasion are probably mediated by decreased transcription of c-Met signaling genes (**Figure 6F, right**).

### THZ1 suppressed tumor growth in a patient-derived xenograft (PDX) model of ICC

**Figure 7:**
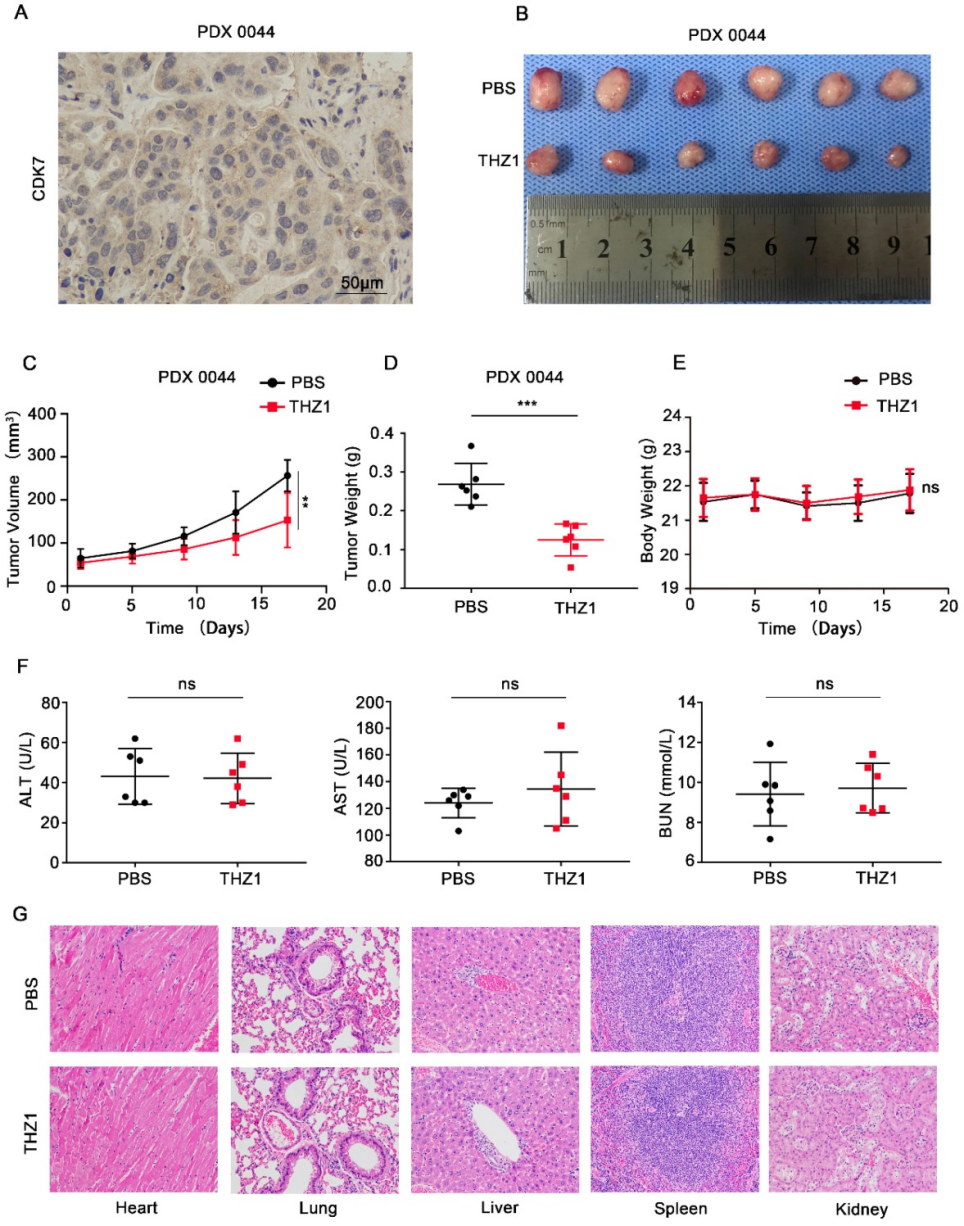
** THZ1 suppressed the growth of patient-derived ICC xenografts.** A. IHC staining of CDK7 in PDX0044 tumors. Scale bar= 50 μm. B. Images of PDX tumors derived from mice (n=6) treated with either PBS or THZ1 (10 mg/kg, twice a day for 17 days). C. Tumor growth curves of mice (n=6) treated with either PBS or THZ1 (10 mg/kg, twice a day for 17 days). Tumor volume was monitored at 4-day intervals. D. Weight of PDX tumors derived from mice (n=6) treated with either PBS or THZ1 (10 mg/kg, twice a day for 17 days). E. Body weight of the mice (n=6). F. Serum ALT, AST, and BUN levels in mice in the different groups. G. Hematoxylin and eosin (H&E) staining of the major organs (heart, lung, liver, spleen, and kidney) of mice in the different groups. The results are presented as mean ± SD of three independent experiments (*P<0.05, **P<0.01, ***P<0.001, according to a Student's t-test).

Next, we investigated whether CDK7 inhibition could suppress tumor growth *in vivo*. PDX models that simulate human tumor biology in rodents are useful tools for evaluating the efficacy of anti-tumor drugs 15. We first generated a murine PDX model of CDK7-overexpressing ICC (PDX0044) (**Figure 7A**). Once the xenografts reached a volume of 50-100 mm^3^, the tumor-bearing mice were randomly divided into two groups, and then intraperitoneally injected with either PBS or THZ1 (10 mg/kg) twice a day for 17 days. THZ1 treatment significantly reduced the tumor volume and weight (**Figure 7B-D**), without eliciting detectable toxic effects. THZ1 did not affect the body weight (**Figure 7E**) or the liver or renal function (**Figure 7F**) of the mice. Furthermore, no structural damage to the major organs (heart, lung, liver, spleen, and kidney) was noted upon THZ1 treatment (**Figure 7G**). Collectively, these results demonstrate that THZ1 has anti-neoplastic effects against ICC *in vivo*.

## Discussion

In this study, we demonstrated that inhibition of CDK7, a highly expressed transcriptional regulator in ICC, has therapeutic effects on ICC tumor growth and invasion. The high potency of THZ1 in ICC can be explained in part by transcriptional repression of oncogenes responsible for uncontrolled cell proliferation and metastasis, specifically those involved in the cell cycle and c-Met signaling. Importantly, we demonstrated that THZ1 treatment (10 mg/kg, twice a day) exhibited no detectable side effects while significantly suppressing the growth of ICC PDX *in vivo*. The selectivity of THZ1 for ICC cells may also be attributed to the disruption of the transcriptional program associated with aberrant cell cycle regulation and activated c-Met signaling pathway, which are commonly overactive in ICC.

Cancer is characterized by uncontrolled cell proliferation caused by aberrant activity of various cell cycle regulators (e.g. CDKs) 16. Therefore, cell cycle regulators are attractive targets for tumor therapy 17. To the best of our knowledge, we are the first to reveal that CDK7 expression is positively correlated with tumor size, TNM stage, and poor prognosis in ICC. These findings suggest that CDK7 level can serve as a prognostic biomarker in ICC patients after surgical resection. Moreover, the results of our ICC PDX model experiment confirm that CDK7 is a viable therapeutic target for ICC therapy. The poor prognosis of patients with ICC is largely due to the high rate of recurrence and metastases after surgical resection. ICC patients with lymph node metastasis are at a high risk of recurrence. In our study, CDK7 inhibition reduced the migration and invasion activity of ICC cells, and suppressed the c‐Met signaling pathway, whose aberrant activation is implicated in metastasis 18. These findings indicate that targeted CDK7 inhibition could be a potential treatment strategy in metastatic ICC. However, more experimental evidence is needed to validate this hypothesis.

Aberrant super-enhancers (SEs) that recruit the transcription machinery to drive high-level expression of genes are found to establish the dysregulated transcriptional program in cancer cells. THZ1 treatment at low dosage mainly affects transcription of genes under the dependency of SEs. Therefore, the specificity of THZ1-sensitive transcripts relies on the specific transcriptional program of SE dependent genes in different types of tumors. Previous studies have shown the effects of THZ1 on transcription regulation and cell-cycle arrest in numerous cancers. For example, Lu et al. reported that CDK7 inhibition resulted in a pronounced downregulation of MYC and NF-κB signaling-related transcripts in PDAC cells 19. However, the results of our RNA-seq analysis showed that c-Met is a critical target of THZ1, which suggests the tumors with high c-Met expression may be particularly responsive to treatment with THZ1 in the clinic. c-Met, also called MET or hepatocyte growth factor receptor, belongs to a subfamily of heterodimeric receptor tyrosine kinases (RTKs)^20^. Previous reports showed that c-Met is overexpressed in 12-58% of ICC tumors. Activation of c-Met signaling triggers a series of biological activities, which are known as the invasive growth program 21, 22. Our results suggest that the c-Met-triggered transcriptional program might be a crucial downstream target of THZ1-mediated suppression of ICC cell invasion. This hypothesis could be tested through an invasion assay with c-Met overexpressing cells treated with either PBS or THZ1. Recently, THZ1 was shown to significantly inhibit MCL1 transcription in cholangiocarcinoma cells 23. However, it did not produce the same invasion-defective phenotype seen in ICC cells, providing further evidence that aberrant c-Met expression may be necessary for the inhibitory activity of THZ1 on cell invasion. The dependency on c-Met rather than on MCL1 may explain why THZ1 selectively kills expanded ICC cells without causing toxic side effects in mice.

Cell-cycle regulatory proteins are often overactive in cancer cells. Because cancer cells are “addicted” to specific CDKs, CDK inhibition can selectively target tumor cells while sparing normal cells 24. Our data suggest that THZ1-induced inhibition of ICC cell growth is mediated by G2/M arrest. In some cases, inhibition of CDK activity not only leads to cell cycle arrest, but also induces senescence or apoptosis of tumor cells. For example, Huang et al. showed that THZ1 in combination with ABT-263 drives apoptosis of cholangiocarcinoma cells 23. In our study, THZ1 blocked transcription of genes regulating the G2/M checkpoint (*AURKA, AURKB, CDC25B, CDK1*), the S-phase regulatory gene (*CCNA2*), and the cell proliferation marker gene (*MKI67*). It remains unclear why the downstream targets of THZ1, and THZ1-induced phenotypes, are different in cholangiocarcinoma. Understanding the mechanism of CDK7 dependent transcriptional program in different subtypes of cholangiocarcinoma may be helpful to select appropriate patients for THZ1 treatment. For example, we showed c-Met is a critical target of THZ1, which suggests the tumors with high c-Met expression may be particularly responsive to treatment with THZ1 in the clinic. Moreover, treatment with THZ1 alone will lead to acquired drug resistance. Combination strategies with drugs targeting different carcinogenic pathways can induce more durable and improved responses.

In summary, our data show that a high level of CDK7 is associated with poor prognosis in patients with ICC. The phenotypic changes induced in ICC by CDK7 depletion or THZ1 treatment indicate that CDK7 is involved in cell proliferation, tumor sphere formation, migration, invasion, and cell cycle regulation. THZ1, a selective covalent inhibitor of CDK7, has shown promise in the therapy of ICC. Thus, we propose that CDK7 is a useful prognostic biomarker and an attractive therapeutic target for ICC.

## Supplementary Material

Supplementary figure and tables.

## Figures and Tables

**Table 1:** Correlation between CDK7 expression and clinicopathological characteristics in 96 ICC patients

Characteristics		Number of patients		*P*-value^*^
		Low CDK7 expression	High CDK7 expression	
Gender	Male	27	26	0.684
	Female	24	19	
Age	≤60	31	22	0.305
	>60	20	23	
Tumor size	≤5cm	25	12	0.035
	>5cm	26	33	
CA19-9, kU/L	≤37	28	17	0.105
	>37	23	28	
TBIL, µmol/L	≤ 34.4	40	38	0.602
	>34.4	11	7	
TNM stage	I/II	29	14	0.014
	III/IV	22	31	
T stage	I/II	38	28	0.27
	III/IV	13	17	
Lymphatic metastasis	Positive	13	18	0.189
	Negative	38	27	
Distant metastasis	Positive	7	9	0.291
	Negative	44	36	
Vascular invasion	Positive	9	8	1
	Negative	42	37	
Nerve invasion	Positive	6	7	0.766
	Negative	45	38	

^*^ Chi-square test.

## Abbreviations

ICC: intrahepatic cholangiocarcinoma
CDK7: cyclin-dependent kinase 7
RNA-seq: RNA-sequencing
HCC: hepatocellular carcinoma
CDK: cyclin-dependent kinase
CAK: CDK-activating kinase
IHC: immunohistochemistrical
siRNAs: small interfering RNAs
OS: overall survival
DFS: disease-free survival
FBS: fetal bovine serum
qRT-PCR: quantitative reverse transcriptase PCR
IC50: half maximal inhibitory concentration
PDX: patient-derived xenograft
ALT: alanine aminotransferase
AST: aspartate aminotransferase
BUN: blood urea nitrogen
H&E staining: hematoxylin and eosin staining
GO: gene ontology
GSEA: gene set enrichment analysis
RTKs: receptor tyrosine kinases
