# Supplementary material for: Therapeutic Targeting of CDK7 Suppresses Tumor Progression in Intrahepatic Cholangiocarcinoma
Source: Int J Biol Sci. 2020 Feb 10;16(7):1207–17. doi: 10.7150/ijbs.39779 (PMC7053328; doi:10.7150/ijbs.39779)
Supplement: Supplementary file 1 — Supplementary figure and tables. [file ijbsv16p1207s1.pdf]

Supplementary Information

Therapeutic Targeting of CDK7 Suppresses Tumor Progression in Intrahepatic Cholangiocarcinoma

Hua-Dong Chen, Chen-Song Huang, Qiong-Cong Xu, Fuxi Li, Xi-Tai Huang, Je-Qin Wang, Shi-jin Li, Wei Zhao, and Xiao-Yu Yin

Contents

Supplementary Figure 1

Supplementary Table 1-2

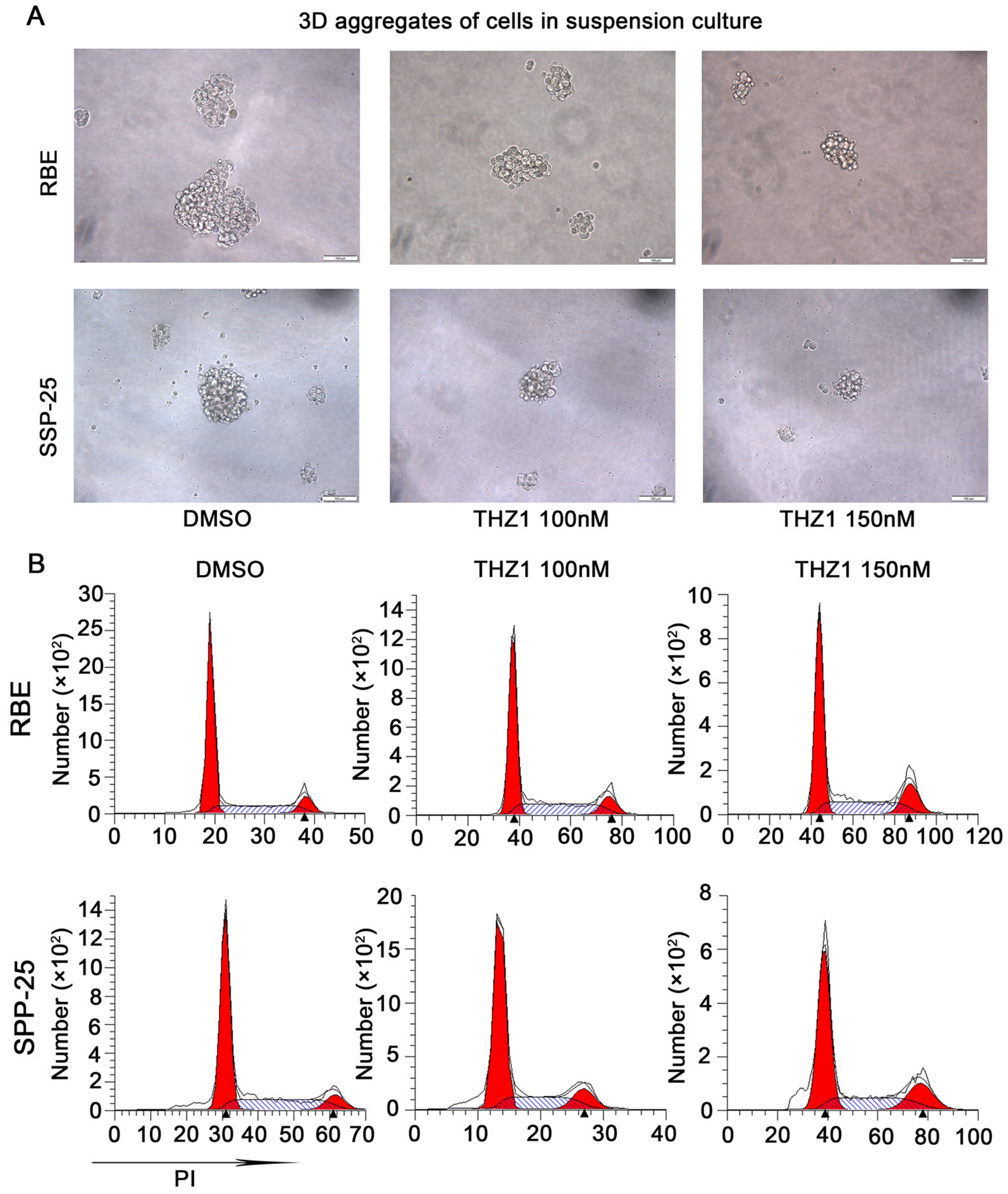

Figure S1. THZ1 inhibited ICC cell sphere formation and induced G2/M cell cycle arrest *in vitro* (related to Figure 4).

A. Tumor sphere formation in RBE and SSP-25 cells upon treatment with different concentrations of THZ1 (100 nM or 150 nM) or 1‰ DMSO on day 5. The representative images were taken on day 7. Scale bar = 100 μm.

B. Cell cycle analysis of RBE and SSP-25 cells upon THZ1 treatment (100 nM or 150 nM) with different concentrations for 48 h.

Table S1. The clinicopathological characteristics of 96 ICC patients

Characteristics	Values
Age, year (mean ± SD)	57.31 ± 11.6 (21-79)
Gender (male/female)	53/43
Tumor size, cm (≤5/＞5)	37/59
CA19-9, kU/L (≤37/＞37)	45/51
TNM stage (I/II/III/IV)	29/14/35/18
T stage (I/II/III/IV)	39/27/4/26
Lymphatic metastasis (Negative, Positive)	65/31
Distant metastasis (Negative, Positive)	16/80
TBIL, μmol/L (≤37/＞37)	78/18
Vascular invasion (Negative, Positive)	79/17
Nerve invasion (Negative, Positive)	83/13
Tumor recurrence, months (mean ± SD)	17.1±22.8 (1-118)
Overall survival, months (mean ± SD)	21.7 ± 22.8 (1-118)

Table S2. Sequences of primers and siRNA used for experiments in this study

Names	Sequences
GAPDH-primer-F	AGATCATCAGCAATGCCTCCT
GAPDH-primer-R	TGAGTCCTTCCACGATACCAA
CDK7-primer-F	ATGGCTCTGGACGTGAAGTCT
CDK7-primer-R	GCGACAATTTGGTTGGTGTTC
CDK7-siRNA-1-sense	GGACAUAGAUCAGAAGCUA
CDK7-siRNA-2-sense	GCUAAGUCAUCCAAAUAUA
AURKA-primer-F	TTGGGTGGTCAGTACATGCT
AURKA-primer-R	CCTGGCTCCCTCTGTTACAA
AURKB-primer-F	TTTGAGATTGGGCGTCCTCT
AURKB-primer-R	ATCACCTTCTTCCCATGGCA
CDC25B-primer-F	AAAGACCTTCCGCCTCAAGA
CDC25B-primer-R	ACCCACACCATCTTTCCCAT
CDK1-primer-F	GGGGTCAGCTCGTTACTCAA
CDK1-primer-R	TGACATGGGATGCTAGGCTT
CCNA2-primer-F	CACTCTACACAGTCACGGGA
CCNA2-primer-R	AGTGTCTCTGGTGGGTTGAG
MKI67-primer-F	AGCTCAAGATTCCAAGGCCT
MKI67-primer-R	CCAGAAATGGGATCAGCTGC

MET-primer-F	CCCACCCTTTGTTCAGTGTG
MET-primer-R	AGTCAAGGTGCAGCTCTCAT
AKT1-primer-F	GCCCAACACCTTCATCATCC
AKT1-primer-R	ACACCTCCATCTCTTCAGCC
PTK2-primer-F	AATCGGCCCAGAAGAAGGAA
PTK2-primer-R	CGCAATGGTTAGGGATGGTG
CRK-primer-F	AAGTATAGACCTGCCTCCGC
CRK-primer-R	AACCTTTACCAGCTCACCGA
PDPK1-primer-F	TTTCAGGACGACGAGAAGCT
PDPK1-primer-R	TCCCTGTGAATGATGCCCTT
ARF6-primer-F	CTGTGGGTTTCAACGTGGAG
ARF6-primer-R	TTATGGCGTCCCTCATCTCC
